# Neuromelanin detection by magnetic resonance imaging (MRI) and its promise as a biomarker for Parkinson’s disease

**DOI:** 10.1038/s41531-018-0047-3

**Published:** 2018-04-10

**Authors:** David Sulzer, Clifford Cassidy, Guillermo Horga, Un Jung Kang, Stanley Fahn, Luigi Casella, Gianni Pezzoli, Jason Langley, Xiaoping P. Hu, Fabio A. Zucca, Ioannis U. Isaias, Luigi Zecca

**Affiliations:** 1 Department of Psychiatry, Columbia University Medical Center , New York State Psychiatric Institute, New York, NY USA; 2 Department of Neurology, Columbia University Medical Center, New York, NY USA; 3 Department of Pharmacology, Columbia University Medical Center, New York, NY USA; 4 The Royal’s Institute of Mental Health Research, Affiliated with the University of Ottawa, Ottawa, ON Canada; 5 Department of Chemistry, University of Pavia, Pavia, Italy; 6 Parkinson Institute, ASST “Gaetano Pini-CTO”, Milan, Italy; 7 Center for Advanced NeuroImaging, University of California Riverside, Riverside, CA USA; 8 Department of Bioengineering, University of California Riverside, Riverside, CA USA; 9 Institute of Biomedical Technologies, National Research Council of Italy, Milan, Italy; 10 Department of Neurology, University Hospital and Julius-Maximillian-University, Wuerzburg, Germany

## Abstract

The diagnosis of Parkinson’s disease (PD) occurs after pathogenesis is advanced and many substantia nigra (SN) dopamine neurons have already died. Now that therapies to block this neuronal loss are under development, it is imperative that the disease be diagnosed at earlier stages and that the response to therapies is monitored. Recent studies suggest this can be accomplished by magnetic resonance imaging (MRI) detection of neuromelanin (NM), the characteristic pigment of SN dopaminergic, and locus coeruleus (LC) noradrenergic neurons. NM is an autophagic product synthesized via oxidation of catecholamines and subsequent reactions, and in the SN and LC it increases linearly during normal aging. In PD, however, the pigment is lost when SN and LC neurons die. As shown nearly 25 years ago by Zecca and colleagues, NM’s avid binding of iron provides a paramagnetic source to enable electron and nuclear magnetic resonance detection, and thus a means for safe and noninvasive measure in living human brain. Recent technical improvements now provide a means for MRI to differentiate between PD patients and age-matched healthy controls, and should be able to identify changes in SN NM with age in individuals. We discuss how MRI detects NM and how this approach might be improved. We suggest that MRI of NM can be used to confirm PD diagnosis and monitor disease progression. We recommend that for subjects at risk for PD, and perhaps generally for older people, that MRI sequences performed at regular intervals can provide a pre-clinical means to detect presymptomatic PD.

## The need to detect Parkinson’s disease (PD) prior to the arrival of symptoms

Following the identification of alpha-synuclein mutations,^1^ variants of numerous genes have been associated with familial PD.^2^ Many of these alleles exhibit low “penetrance”, as many who carry these variants never develop PD. There are moreover environmental associations with PD, including pesticide or manganese exposure,^3,4^ but the majority of exposed individuals do not develop PD.

PD diagnosis occurs only when the motor symptoms are apparent and 30% or more of the substantia nigra (SN) dopamine neurons have already died.^5^ This presents a fundamental challenge to clinical treatment: while there are multiple promising therapies under exploration, including the use of growth factors,^6^ ion channel ligands,^7^ immune and gene therapies, antioxidants, and exercise regimes, such prophylactic approaches should begin early in the disease course, before many neurons are sick or dead.

No useful test exists to diagnose PD prior to the arrival of motor symptoms. PD patients often exhibit early disease-associated features, including loss of smell, restless leg syndrome, anxiety, depression, and constipation and REM sleep behavior disorder, but these are associated with many disorders. A high proportion of individuals with rapid eye movement sleep behaviors disorders (RBD) develop synucleinopathies, but the progression occurs over decades. Moreover, not all people who develop PD have a prior history of RBD. It is widely suspected that damage to dopaminergic axons may precede cell death, and single-photon emission computed tomography (SPECT) or positron emission tomography (PET) imaging for dopamine axon-associated labels such as the dopamine transporter (DAT) or vesicular monoamine transporter 2 (VMAT2) are promising, but these are expensive and involve radiation exposure.^8^ Assays of PD-specific immunological responses, including the presence of specific T cells populations,^9^ are promising, but as recently reviewed,^10^ the use of candidate cytokine markers may not provide adequate differentiation between multiple disorders.

An alternate approach is to examine the targeted neurons. The presence of the dark neuromelanin (NM) pigment provides the names “SN” and “locus coeruleus” (LC). About a century after the first description of PD,^11^ the disease was noticed to feature the loss of the NM pigmented cells of the SN.^12^ These pigmented neurons were eventually found to be dopaminergic.^13^ A similar loss occurs during PD of the pigmented noradrenergic neurons of the LC (for a detailed review of targeted neurons in PD, see ref. ^14^).

It took nearly another century to develop a means to detect these pigmented neurons in living patients. About 15 years ago, Zecca, Sulzer, and colleagues^15^ suggested from their measurement of NM concentration in SN of normal and PD subjects that “NM appears to be a good marker of damage to the SN pars compacta (SNc) in PD. As such, the development of NM in vivo imaging techniques may offer diagnostic and disease staging measurements matching the available tools used to monitor striatal dopamine depletion and offering the first direct approach to recognize and quantify nigral damage. The concentration values of NM here described could be used for correlation with in vivo images of SN in normal and PD subjects. Further studies should expand the above observations in other extrapyramidal disorders to assess specificity of NM depletion and correlation with other established markers of nigrostriatal dysfunction”.

Since that time, efforts by the authors and others have introduced a magnetic resonance imaging (MRI) approach that promises a means to assay individuals on their way to developing the disorder before most of these neurons have died. The current results are promising, but much work remains before this approach is adapted to the clinic. We discuss the rationale, theory, practice, and advantages and disadvantages of this approach, and offer suggestions on future research and potential use in therapy.

## NM pigment is synthesized from oxidized catecholamines that are trapped within autophagic organelles

There are parallels between NM and the melanin pigments of skin, hair, eyes, feathers, and squid ink, as both are produced as oxidative products downstream from L-DOPA, and both classes of pigments play protective roles against damage to the cytosol, in the case of melanin by decreasing photodamage. Such catecholamine oxidation is a common way to produce pigment in biology: for instance, the dark pigment that occurs in bananas is the product of an enzymatic oxidation of dopamine.^16^

The “true” melanins are produced in specialized lysosomes known as melanosomes in specialized cells known as melanocytes via a specific enzyme, tyrosinase, that is nearly absent in mature human brain and is not found in SN.^17,18^ The melanosomes are secreted and then acquired by the pigmented cells.

**Fig. 1:**
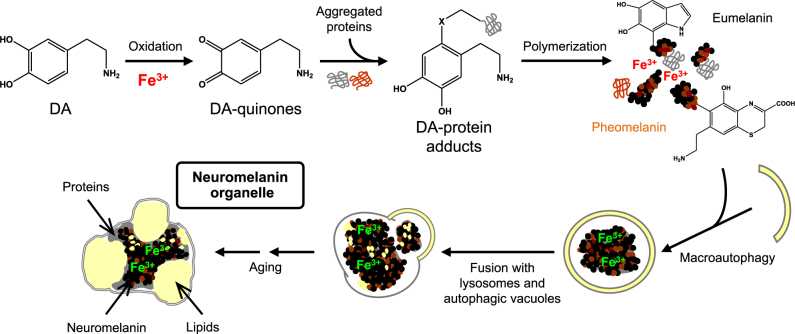
Mechanisms for biosynthesis of NM pigment and for the formation of NM-containing organelles in human SN. Excess dopamine in the cytosol of SN neurons can be oxidized to quinones by ferric iron. These highly reactive compounds can bind to aggregated and β-structured proteins that accumulate in the cytosol. An oxidative polymerization initiates formation of the melanin-protein component with eumelanin and pheomelanin moieties that can also bind high amounts of metals, particularly iron. Via macroautophagy, the resulting undegradable material is taken into autophagic vacuoles that fuse with lysosomes and other autophagic vacuoles containing lipid and protein components, thus forming the final NM-containing organelles that contain NM pigment along with metals, abundant lipid bodies, and protein matrix. The process continues during the life of the neuron, so that SN dopamine neurons accumulate high numbers of NM-containing organelles with age. This scheme is based on characterizations by multiple techniques of lipid and protein systems in NM-containing organelles of the human SN (Zucca et al., under review). Figure modified from ref. ^20^ by permission of Springer and ref. ^21^ by permission of Elsevier

NM uses a very different synthetic pathway (Fig. 1). The color and electron dense property of NM pigment is derived from the conversion of dopamine, norepinephrine, and other catecholamines in the cytosol by oxidation to semi-quinones and quinones.^19^ While tyrosinase is absent, other enzymes might be involved in NM synthesis (for an extensive review of possible enzymatic participation in NM synthesis, see refs. ^20,21^). The catechol oxidation reaction may also occur non-enzymatically, and iron and other redox metals strongly promote the auto-oxidation of catecholamine to quinones.^19^

**Fig. 2:**
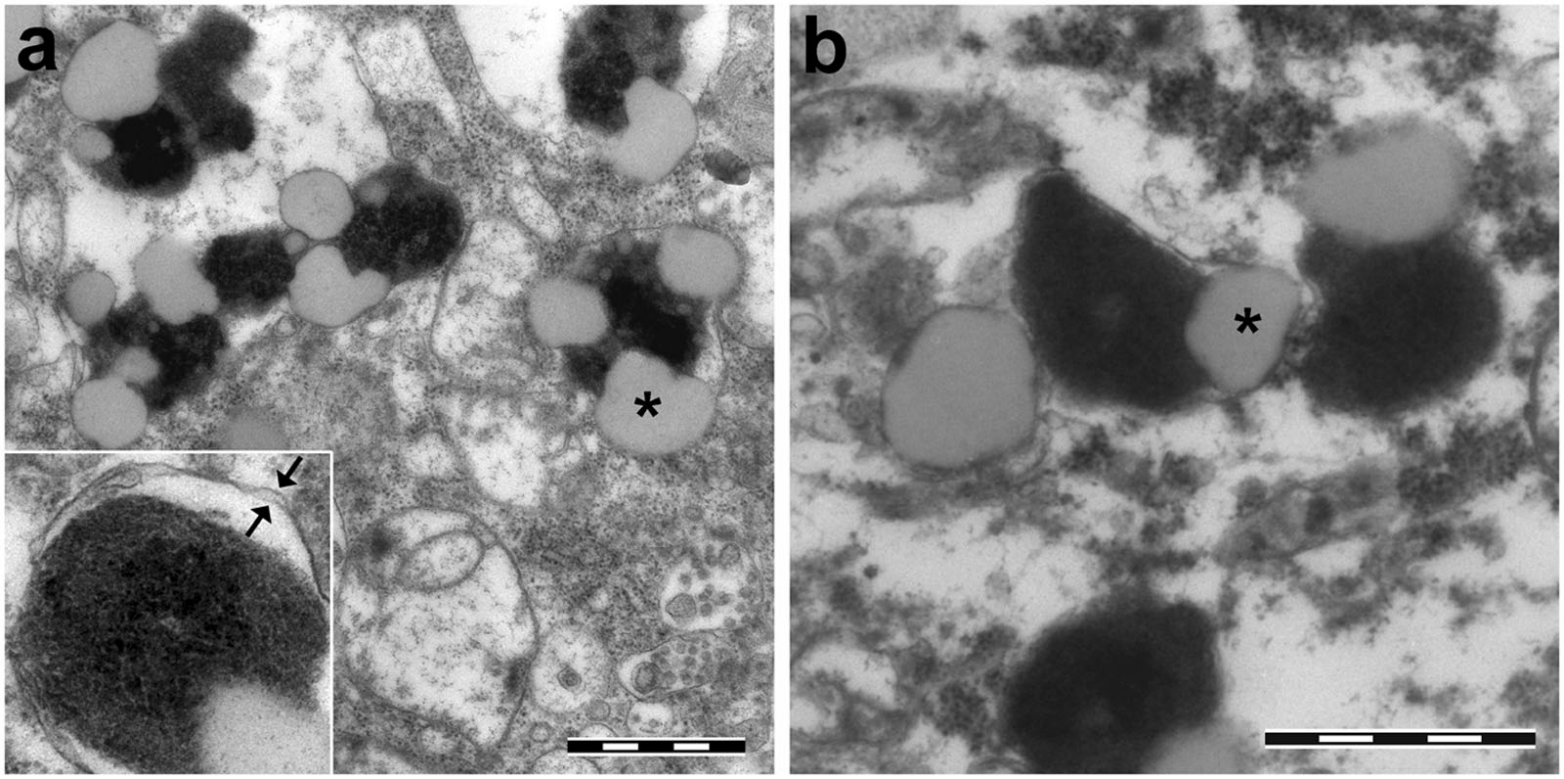
Transmission electron microscopy images of NM-containing organelles of human SN (**a**) and LC (**b**). These specialized autophagic vacuoles display high amount of NM pigment (black and electron dense), a protein matrix and lipid droplets (asterisk). The intraneuronal NM-containing organelles can often be observed to possess double membranes, consistent with macroautophagy. The inset of **a** shows a single NM-containing organelle in SN with a double membrane delimiting the organelle (arrows). **a** SN tissue from 78 y.o. healthy subject. Scale bar = 1 µm. **b** LC tissue from 81 y.o. healthy subject. Scale bar = 1 µm. For tissue treatments and ethics policies, refer to refs. ^24,32^ Figure reproduced from ref.^25^ by permission of John Wiley and Sons

NM pigment, while probably initially produced in the cytosol, is accumulated within autophagic organelles.^22^ In this macroautophagic pathway, multilamellar organelles known as autophagosomes envelop intracellular constituents and traffic to lysosomes, with which they fuse (Fig. 1). Broadly, autophagosomes are produced in response to many forms of cellular stress, apparently including high levels of cytosolic dopamine, and so the presence of NM organelles may be both a sign of oxidative stress and a protective response to it. These NM-containing organelles possess abundant lipid bodies and soluble and pigment bound proteins (Fig. 2).^23,24^

Notably, lipofuscin and ceroid pigment organelles also originate in a similar manner, although their morphology differs from that of NM-containing organelles.^25^ In each case, the lysosomes do not efficiently break down the pigments and they are accumulated with age, unless the cells die, in which case specialized phagocytic cells such as microglia or macrophages, which have far more powerful degradation systems than neurons, destroy NM organelles and the NM pigment itself; this process can be observed by video microscopy when microglia are exposed to NM.^26^

The formation of NM-containing organelles appears to play a protective role in neurons, as excessive dopamine in the cytosol induces damage by reactive oxygen species that can cause neuronal death.^27^ In neuronal cell culture, overexpression of the VMAT2 enhances dopamine accumulation by synaptic and secretory vesicles^28^ and so decreases cytosolic dopamine levels and protects neurons from death,^27^ and also decreases NM formation.^22^ In human brain, one study identified a correlation between high levels of VMAT2 expression and low NM in midbrain dopamine neurons.^29^

Within the autophagic organelles, NM pigment consists of granular aggregates of 200–400 nm composed of smaller spherical substructures of ~30 nm diameter^24,30,31^ that form spherical electron-dense aggregates. In NM, the melanic components are covalently bound to peptides that have cross-β-sheet structure and aliphatic chains derived from dolichols and dolichoic acids (polyisoprenic compounds). The dolichols and dolichoic acids^32^ are the major consitituents^23,24,33–35^ of unpigmented “lipids bodies” within the NM-containing organelle.

The cross-β-sheet structured fibrillar peptide in the NM-containing organelle constitutes a seed around which the unstructured melanic component of NM progressively grows, upon conjugation of dopamine quinone units or oligomers in a stepwise process. This is why, in contrast to other melanin pigments, NM lacks the aromatic stacking organization typical of the polymers derived from tyrosinase oxidation of phenolic and catecholic substrates. The only structural motif recognized by X-ray powder diffraction in NM pigment is related to the cross-β protein/peptide core, and not to the melanic component.^21^

The formation of the melanin-protein conjugates during the early steps of NM formation has been replicated in the synthesis of similar conjugates containing fibrillar protein cores.^36^ As detailed in the section below, “The NM–iron complex is paramagnetic and detectable by EPR”, the melanic component of NM contains a stable free radical, as electron paramagnetic resonance (EPR) demonstrates the presence of an organic radical species associated with the catechol moieties (likely a semi-quinone), as well as high spin iron(III), and the two paramagnetic species closely interact. This is consistent with the hypothesis that sequestering these compounds in an autophagic organelle away from the cytosol provides a protective mechanism.

Recent proteomic analysis of NM-containing organelles demonstrates the presence of many proteins. These include as expected, lysosomal matrix and membrane-associated proteins, and autophagic proteins (Zucca et al., under review). Particularly notable components, due to their association with PD, are alpha-synuclein, which is aggregated in virtually all PD cases, and the antigen presenting protein, major histocompatibility complex class I, which appears to be more highly expressed in adult SN dopamine and LC norepinephrine neurons than other brain neurons.^37^

In summary, NM is an insoluble pigment originating from dopamine-derived quinones contained within autophagic lysosomes together with lipid bodies and many soluble proteins (Fig. 1). In NM, the melanic component is bound to cross-β-sheet peptides and aliphatic chains derived from dolichols, that along with dolichoic acids are the major components of lipid bodies within the NM-containing organelle. NM binds high levels of iron and other metals, forming a paramagnetic complex crucial for MRI detection. The synthesis of NM is thought to be neuroprotective as it removes excess cytosolic dopamine.

## Iron and NM interactions

NM pigment chelates transition metal ions, including iron, copper, zinc.^24,38,39^ These metals can participate in catecholamine oxidation during the early steps of NM synthesis and remain bound to NM in the autolysosomal organelle.^21,39^ They also are responsible for the ability of MRI to detect NM.

Iron, the fourth most abundant element in the earth’s crust, is the most abundant metal in NM pigment, and NM provides by far the highest levels of iron in pigmented SN dopamine neurons.^21^ In the SN of healthy subjects, the tissue concentration of iron is about 0.1–0.25 μg/mg, while the iron concentration in intraneuronal NM is about 6 μg/mg of NM pigment, and the iron levels of isolated NM pigment is ~11 μg/mg.^40–42^ These values suggest that intraneuronal NM is unsaturated and has the capacity to chelate more iron and other metals. The concentration of iron in NM pigment isolated from LC is only 16% of that in NM from SN,^43^ but LC NM binds more copper than SN NM, perhaps due to the presence of copper as a cofactor for dopamine beta-hydroxylase for the synthesis of norepinephrine.^41^

Iron ions are highly reactive, and can produce the hydroxyl radical via the Fenton reaction. Iron complexes in biology generally consist of ferrous (Fe^2+^) and ferric (Fe^3+^) iron, often in redox equilibrium, chelated with small molecules such as citrate or ATP, and proteins including hemoglobin, cytochromes, ferritin, lactoferrin, and transferrin. Tyrosine hydroxylase, which typically provides the rate-limiting step in dopamine synthesis, uses iron as a cofactor,^44^ while as above, in norepinephrine system copper is cofactor for dopamine beta-hydroxylase.

**Fig. 3:**
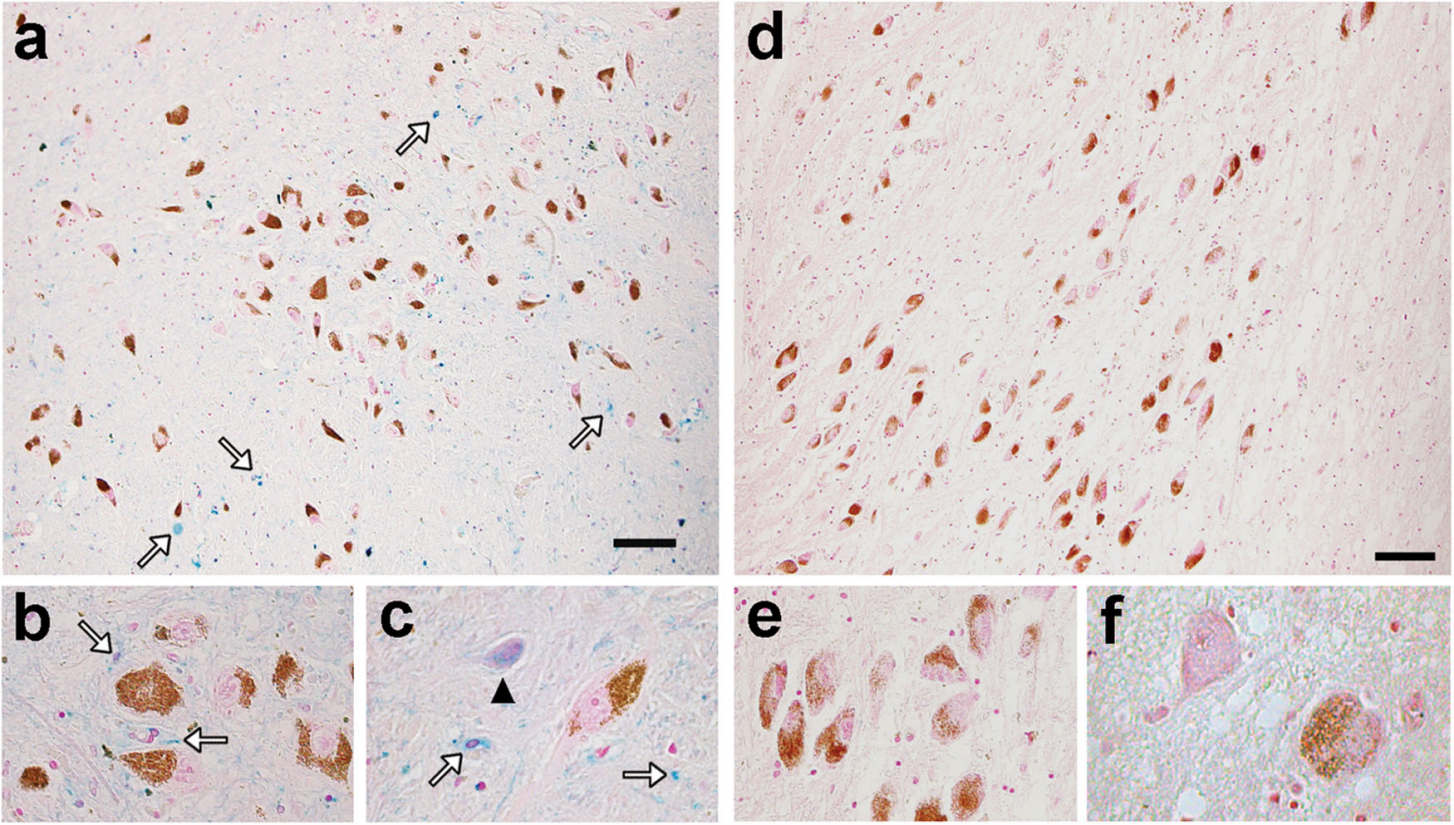
Catecholamine neurons and reactive ferric iron deposits in human SN (**a**, **b**, **c**) and LC (**d**, **e**, **f**) of healthy subjects detected by modified Perls’ staining (for details, see ref. ^41^) NM pigment in SN dopaminergic neurons and LC noradrenergic neurons appears as brown granules and reactive ferric iron deposits are blue. **a**, **b**, **c** In the SN (88 y.o.), many iron deposits are present in whole SN parenchyma and principally contained in glial cells (arrows in **a**, **b**, **c**). As shown in the two panels at higher magnification (**b**, **c**), iron deposits are absent in NM-containing neurons of SN, but non-pigmented neurons show abundant cytoplasmic deposits of reactive ferric iron (arrowhead in **c**), consistent with the ability of NM to scavenge iron in stable complexes. Scale bar = 100 µm. **d**, **e**, **f** In LC (80 y.o.), a light stain was detected in very few glial cells and was completely absent in pigmented and NM-free neurons, as shown at higher magnification panels (**e**, **f**). Scale bar = 100 µm. For tissue treatments and ethics policies, refer to refs. ^24,32^ Figure reproduced from ref. ^138^ with permission from the Royal Society of Chemistry

**Fig. 4:**
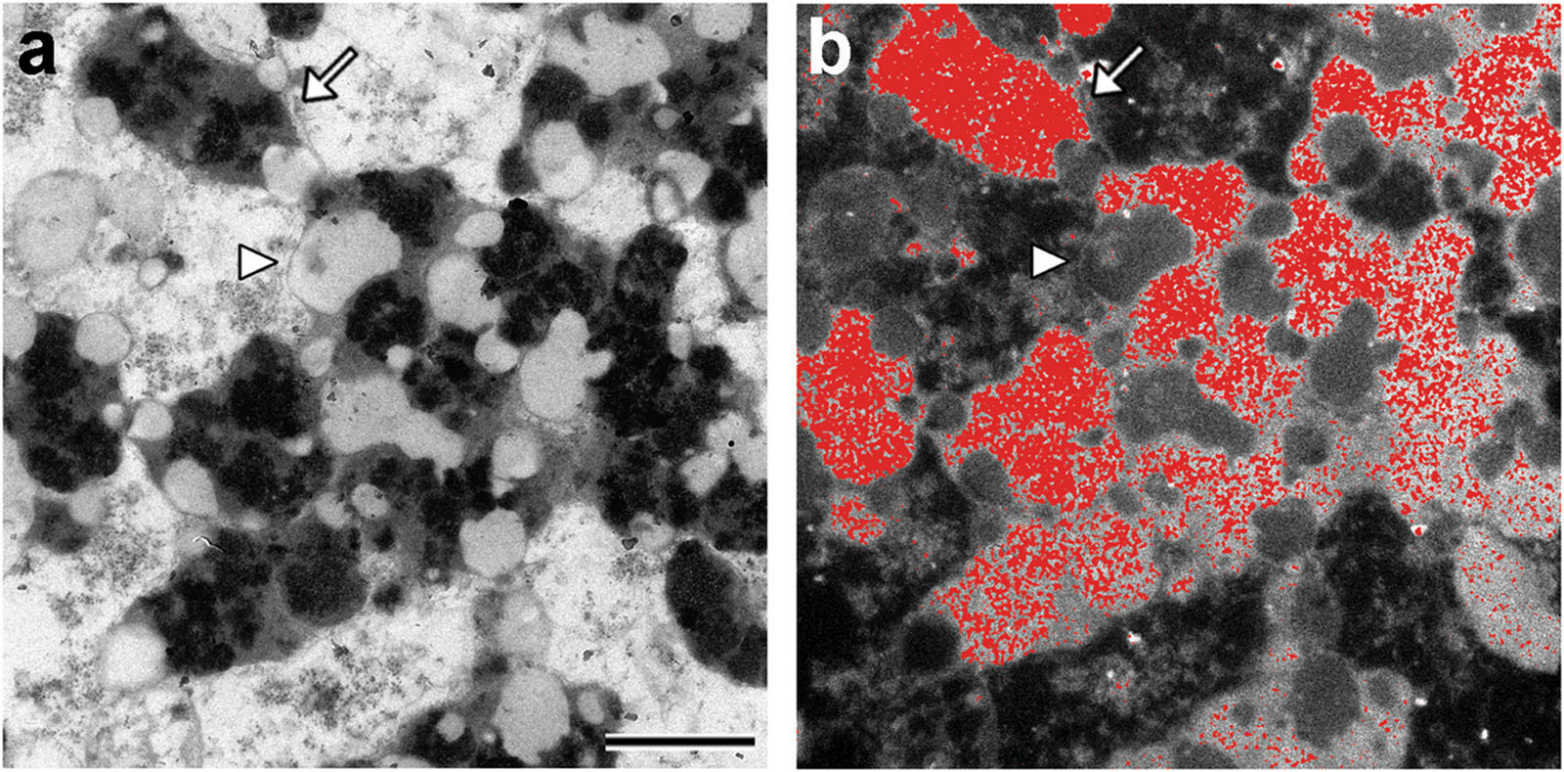
Iron distribution in NM-containing organelles of human SN (89 y.o.) revealed by using electron spectroscopic imaging. In the left panel (**a**) transmission electron microscopy indicates the classical morphology of NM-containing organelles: these organelles contain large amount of dark NM pigment (arrow) strictly associated with lipid bodies (arrow head). The iron distribution map was created by electron spectroscopic imaging (**b**) and revealed that large amounts of iron (red spots) are localized into the NM pigment of the organelles, consistent with the ability of NM pigment to scavenge iron forming stable complexes. Electron spectroscopic imaging were performed using a LEO 912AB electron microscope.^139^ For tissue treatments and ethics policies, refer to refs. ^24,32^ Scale bar = 1 µm

**Fig. 5:**
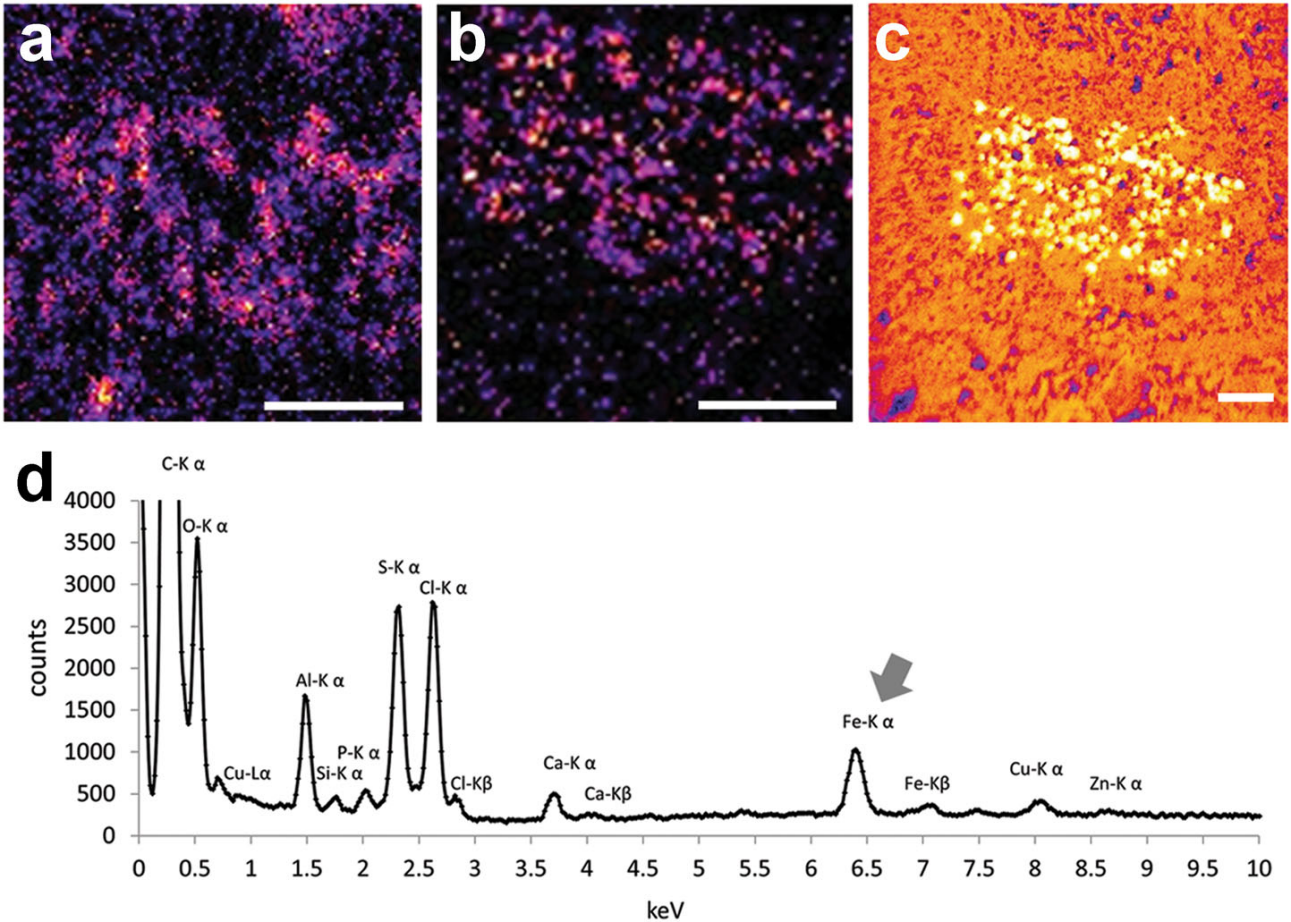
**a**, **b**, **c** Nano-secondary ion mass spectrometry images of human SN. With this technique, a detailed distribution map of iron (**a**), copper (**b**), and sulfur (**c**) was acquired in an SN region highly enriched with NM-containing organelles. Here, the NM pigment of organelles is distinctly traced by sulfur map, due to the presence of this element in the melanic structure of the pigment. Scale bar = 5 µm. **d** Energy-dispersive X-ray microanalysis spectrum of NM pigment within SN organelles. The spectrum is characterized by a large peak of iron (arrow) and smaller peaks of other metals. Figures reproduced from ref. ^46^ by permission of John Wiley and Sons

In healthy aged SN, many deposits of reactive ferric iron are detected by histochemistry in glia and non-NM neurons (Fig. 3a–c), but reactive ferric iron deposits are undetectable in pigmented SN neurons due to efficient NM sequestration.^41,45^ Although reactive iron deposits are clearly abundant in the whole parenchyma of SN, these are almost undetectable in LC (Fig. 3d–f) which has lower total iron content than SN.^41^ This binding to NM blocks the redox activity of the iron, and likely prevents neurotoxicity.^39,42^ This localization of iron within the NM organelle has been confirmed by electron spectroscopic imaging (Fig. 4) and by analytical electron microscopy and nano-secondary ion mass spectrometry (Fig. 5).^21,46^

**Fig. 6:**
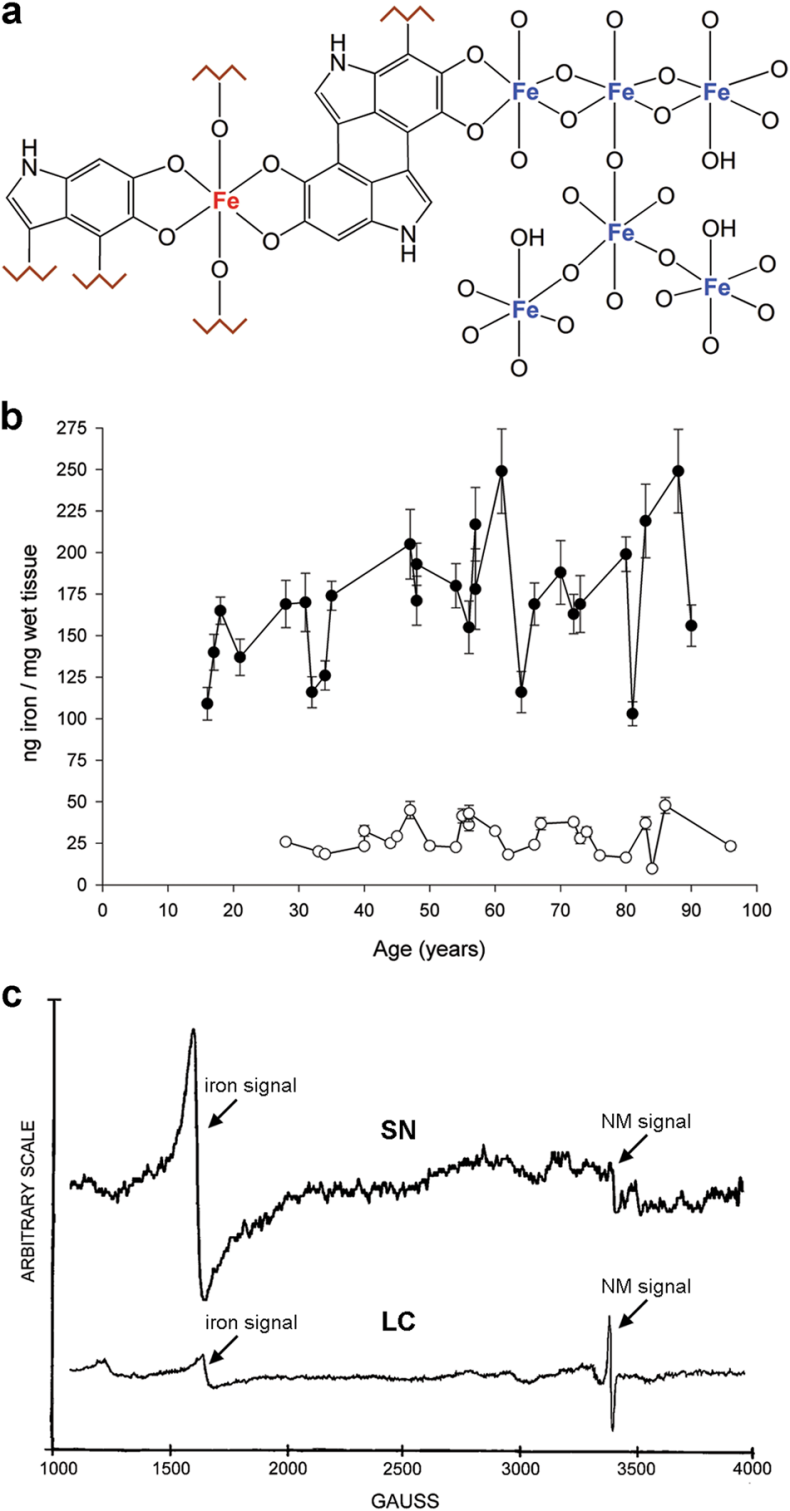
**a** Two distinct iron-binding sites are present in the human NM pigment with different affinity. The multinuclear iron cluster, similarly to ferritin, contains iron(III) ions (blue colored) that are coupled by oxy-hydroxy bridges and bound through catechol groups to melanic portion of NM pigment. At this site, iron is likely stored with high affinity and maintained in a redox inactive state, and is principally detected by Mössbauer spectroscopy. In the mononuclear iron center, iron ions (red) are six-coordinated by oxygen atoms of catechols moieties in octahedral arrangement, and possibly by hydroxo groups. This could be a low-affinity binding site occupied in cases of iron overload, when the high-affinity centers are saturated, as occurs in iron overload conditions of PD. In this case, the mononuclear iron could be redox reactive and catalyze the production of toxic species causing iron-mediated toxicity. Iron in this site is principally detected by EPR spectroscopy. The structural differences between high and low-affinity sites, their accessibility, and reactivity toward small binding ligands and biological substrates, with reliable quantification of iron, still need further investigation. Figure reproduced from ref. ^21^ by permission of Elsevier. **b** Concentration of total iron in LC (empty circles) and in SN (black circles) from human normal subjects during aging (mean ± SEM; *n* = 2). The concentration of iron in LC is constant during aging and much lower than that measured in SN tissues. Conversely, the concentration of iron in SN has a smooth increase throughout life according to a linear model. **b** Reproduced from ref. ^41^, copyright (2004) National Academy of Sciences, USA. **c** EPR spectra of NM–iron complex in SN and in LC tissues. The signal at *g* = 4.3 corresponds to iron(III) high spin complex in octahedral configuration, while signal at *g* = 2.0 corresponds to the stable organic radical typically present in both NM pigments and all melanic pigments. From the ratio between two signals intensities and previous EPR calibrations,^40^ it appears that iron content of NM pigment in LC neurons is about 7.9% of that in SN neurons. The same signals were present in NM pigments chemically isolated from LC and SN areas, but with different signal ratios. **c** Modified from ref. ^41^, copyright (2004) National Academy of Sciences, USA

Two distinct ferric iron-binding sites are found in NM pigment (Fig. 6a). At the higher-affinity site, ferric ions are bound by oxy-hydroxy bridges in multinuclear complexes similar to those present in ferritin.^47–50^ In the lower-affinity site, high spin iron(III) interacts with the free radical derived from catechol moieties and is bound as mononuclear, six-coordinated centers of rhombic symmetry.^40,51–55^

As with the trapping of oxidized forms of reactive dopamine within autophagic organelles, the chelation of ferric iron within NM organelles is apparently neuroprotective, and may prevent iron-mediated neurodegeneration by apoptosis or ferroptosis.^56^ While the formation of NM-containing organelles is likely protective in living neurons, after neuronal death, iron bound to NM pigment could become extracellular and lead to toxicity by activating microglia and driving inflammation.^26^

As detailed in the section below, “The basis of MRI”, an important aspect of the NM–iron complex is that it is paramagnetic. Copper, manganese, and other metals can also form paramagnetic NM complexes with similar behavior.

## Changes in NM and iron molecular content during normal aging and PD

**Fig. 7:**
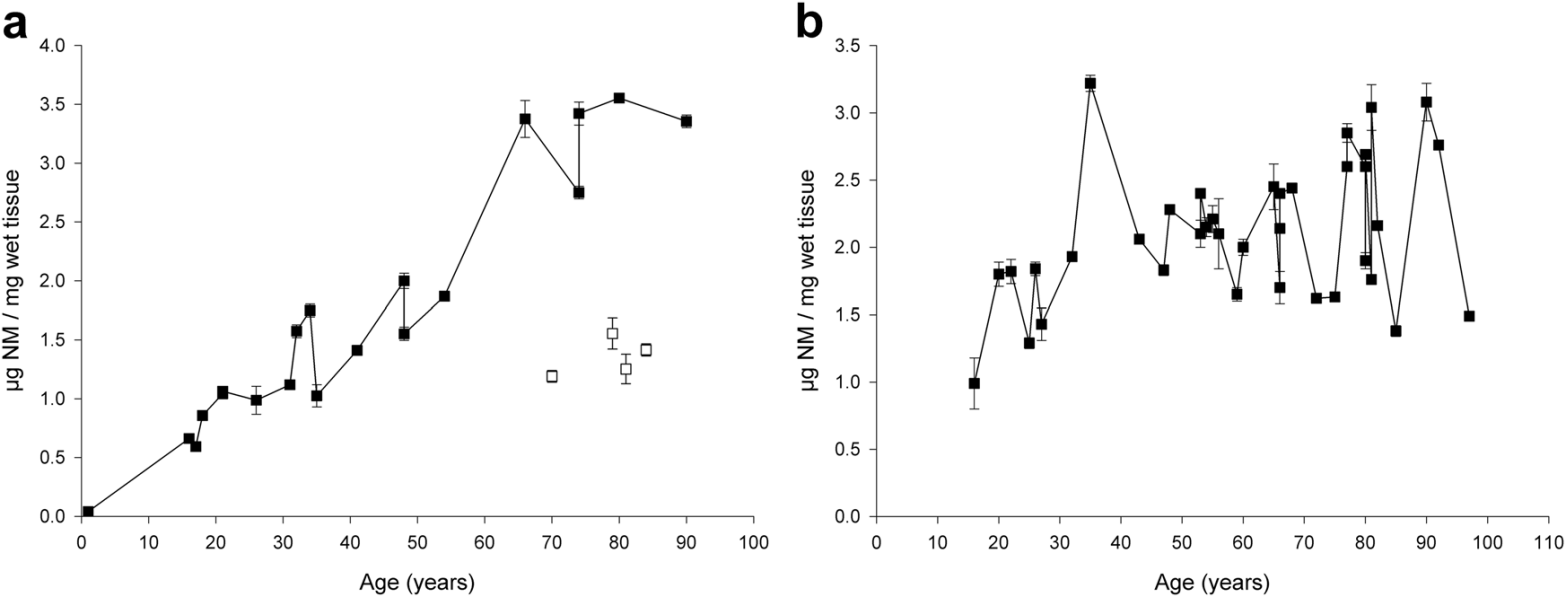
NM pigment concentration in SN tissues of healthy subjects and PD patients (**a**) and in LC tissues (**b**) of control subjects during aging. In the SNc (**a**) of normal male subjects, the NM content increases during life linearly (black squares). Four PD subject (empty squares) had far lower NM concentration in their SNc compared to the age-matched controls (mean ± SEM; *n* = 3–5). Normal male and female subjects had very similar concentration of NM in the corresponding age ranges.^15^ This 50–60% average decrease of NM concentration chemically measured in SNc of PD patients agrees with the histologically detected neuronal loss of SNc pigmented neurons during PD. Figure reproduced from ref. ^15^ by permission of John Wiley and Sons. **b** In LC, the concentration of NM pigment linearly increases during normal aging in both male and female subjects, reaching values similar to those of SN, although the accumulation rate in LC is slower than in SN (mean ± SEM; *n* = 2). Figure modified from ref. ^41^, copyright (2004) National Academy of Sciences, USA

NM pigment in the SN is not visible under light microscopy in newborns, becomes visible at 2–3 years of age^57^, and continues to darken through the 10th decade of life.^15,41,58^ The number of NM-containing neurons during normal aging as measured with histochemical methods has been controversial, with reports of both age-dependent loss and no variation in the number of pigmented neurons.^59–62^ This was readdressed by the development of a chemical method for measuring the absolute concentration of NM in SN by isolating the pigment and measuring UV absorption of solubilized NM.^15^ This approach demonstrated a linear increase with age (Fig. 7a): the average increase rate of NM concentration in SN during aging was estimated as ∼41 ng/mg SN wet tissue per year.^41^ NM concentration in PD SN is often ~50–60% the level of age-matched control subjects, due to the degeneration of neurons containing NM (Fig. 7a).

The total concentration of iron also increases during aging in human SN (Fig. 6b), putamen, globus pallidus, caudate nucleus, and cortex.^41,63,64^ The highest iron content is found in putamen, globus pallidus, and caudate nucleus, with lower concentrations in cortical gray matter, white matter, midbrain (including the SN), and cerebellum, and the lowest iron concentrations are found in the pons, LC, and medulla.^39,41,64–67^

Age-dependent changes also occur in the various molecular forms of iron, including ferritin, NM, transferrin, and hemosiderin, and in the distribution of iron compounds between neurons and glia.^41,58,68^ Ferritin is the major iron molecule in most brain cells. During aging in SN, the total iron (Fig. 6b), H-ferritin, L-ferritin, and NM concentrations (Fig. 7a) increase, while in LC only NM increases (Fig. 7b) and ferritins and iron (Fig. 6b) are lower than in SN and remain constant during the entire lifespan.^41^ In PD, there is an increase of total iron concentration in SN associated with elevated iron bound to the low-affinity sites of NM and likely formation of a redox active complex.^42,69,70^

A very early reported phenomenon, confirmed in many studies, is the presence of extracellular NM in PD patient SN, often in patterns that resemble the extranuclear distribution of the organelles when the neuron was intact.^71,72^ Remarkably, the very early study by Foix and Nicolesco also reported the presence of microglia in PD SN (microglia had only been discovered 6 years prior to their publication^73,74^) that are responsible for the disappearance of the NM following neuronal death. Microglia have been shown in video microscopy to phagocytose NM and, once taken up, rapidly degrade the pigment in minutes in cell cultures.^26^ During this process, the microglia release very high levels of hydrogen peroxide,^26^ which can oxidize and degrade NM as well as pro-inflammatory factors triggering neurodegeneration. This degradative process, however, liberates NM-bound iron and other metals and toxins accumulated during life, which could exacerbate the disease.

## The NM–iron complex is paramagnetic and detectable by EPR

Paramagnetic compounds possess at least one unpaired electron, and such substances have electrons that possess one of two spin orientations.

EPR, also known as electron spin resonance, spectroscopy is a non-destructive method that can be used in intact brain tissue, but the microwave electromagnetic radiation used, close to that in home microwave ovens, prevents its use in vivo. It is highly valuable for providing data on NM structure and its interaction with iron within neurons.

The development of EPR was due to efforts by the Russian scientist E.K. Zavoisky, who initially had intended to develop nuclear magnetic resonance (NMR).^75^ In the presence of an external magnetic field with a given strength, the unpaired electron spin magnetic moment aligns itself either parallel (*m*_s_ = −1/2) or antiparallel (*m*_s_ = +1/2) to the field. Each alignment of spin has a specific energy (*E*) state according to the Zeeman effect:

$$E = m_{\mathrm{s}} g_{\mathrm{e}} \mu _ {\mathrm{B}} B_0,$$

where *B*_*0*_ is the strength of the magnetic field, *g*_e_ is a constant of proportionality, with a value characteristic for an electron of particular compounds,* μ*_B_ is the Bohr magneton, a physical constant. For unpaired free electrons, the separation between upper and lower energy state is given by:

$$\Delta E = g_{\mathrm{e}} \mu _{\mathrm{B}} B_0.$$

At this value, a transition of electrons to the upper energy level and change of electron spin occurs. According to the Maxwell–Boltzmann distribution, the lower energy level is more populated than the upper level. An unpaired electron can move between the two energy levels by absorbing or emitting a radiation with energy of frequency

$$h\nu = \Delta E.$$

Thus,

$$h\nu = g_{\mathrm{e}} \mu_{\mathrm{B}} B_0.$$

The final absorption of energy is measured and converted into a first derivative spectrum, plotted as arbitrary scale of absorbed energy vs. magnetic field strength, which provides the frequency value and thus the *g* value, which can be used to identify the paramagnetic center of the compound.

EPR measurements of NM were introduced by Zecca and Swartz in 1993.^51^ The spins of unpaired electrons on the semiquinone form of the catechol ring in NM provide a magnetic center detectable with EPR. Typically, EPR spectroscopy for NM and melanins are conducted by exciting the sample with at a fixed microwave frequency in the range 9–10 GHz and increasing magnetic field strength between 1000–4000 G (0.1–0.4 T) to reach the Δ*E* value of resonance conditions.

The EPR spectra of isolated NM pigments typically display signals at two *g* values. The first, at *g* = 4.3, is characteristic of ferric iron coordinated by oxygen atoms in the mononuclear, six-coordinated centers of rhombic symmetry.^39,40,51,53,76^ The second signal is at *g* = 2.0 and is due to the stable organic radical typical of melanins.^24,51–53^ In intact SN and LC tissue, EPR spectra show an additional signal at *g* = 6.0 corresponding to ferric heme iron present in different molecules (Fig. 6c). Studies with iron chelation and EPR show exchanges in intensity between the two signals at *g* = 4.3 and *g* = 2,^39,40,53,76^ confirming an interaction between the two paramagnetic centers, and that EPR-detectable iron is bound to the oxygen atoms of the catechol rings.

## The basis of MRI

MRI is also based on paramagnetic resonance, but detects changes at the atomic nuclei via NMR. This occurs at about 2 orders of lower energy excitation than EPR, at radio frequencies of 60–800 MHz, lower than the electromagnetic radiation of a cell phone, and of far less energy than the frequencies used in EPR, which are similar to a microwave oven. Thus, NMR can be used in living brain.

NMR was first described and developed by Isidor Rabi and colleagues.^77^ As with electrons, the neutrons and protons in nuclei also exhibit spin. If the spins of even numbers of protons and neutrons in an atomic isotope pair up to produce a net spin of zero, this produces no magnetic moment, and so these nuclei are invisible to NMR. Many common isotopes, like 1-H and 13-C, have non-zero nuclear spin and are detectable by NMR. Many iron-containing biological compounds can also be studied by NMR, in spite of the presence of unpaired electrons.^78^

With NMR, the nuclear particles are aligned to their lower energy spin state by a constant and strong (to date, most often 3 Tesla in NM-MRI) magnetic field, known as *B*_0_. The alignment is then perturbed by an intermittent pulsed second electromagnetic field applied at radio frequencies known as *H*_0_ that is perpendicular to the direction of *B*_0_. The resonant absorption by the paramagnetic nuclei occurs when, as in EPR, the total energy is at a specific frequency that matches the energy difference between the spin states for a particular substance.

Measurements provided in NMR are typically reported by measuring a time, *T*, for the return of the nuclei to their lower energy spin state in the constant *B*_0_ field following the intermittent *H*_0_ pulse. These are analyzed with two common approaches.

The *T*_1_ signal, also known as “longitudinal relaxation” or “spin-lattice relaxation”, is the mean time for the exponential recovery [1−(1/*e*) = 63%] of the nuclei to recover to the low-energy spin states established by the constant magnetic field. Typical *T*_1_ signals are in the range of 0.4–1.2 ms in water-based tissue and 100–150 ms in fatty tissue.

Alternatively, when the *H*_0_ pulse is not active, the spins related to the perpendicular field are in both states. The *H*_0_ pulse temporarily aligns them, and the *T*_2_ signal is the mean time of the exponential decay [1/*e* = 37%] of the initial signal back to the baseline mixture of spins. The *T*_2_ signal is also known as “transverse relaxation” or “spin–spin” relaxation. *T*_2_ signals for protons are in the range of 40–200 ms in water-based tissue and 10–100 ms in fatty tissue.

In MRI, the paramagnetic particles typically measured are protons. Protocols can be designed as “*T*_1_ weighted images” by allowing enough time for the magnetization to recover after the *H*_0_ pulse, and so uses long periods between the radio pulses, known as the “repetition time” (TR). “*T*_2_ weighted images” use shorter TR and sample the recovery radio “echo” signal soon after the radio pulse, a period known as the “echo time”.

## Contrast mechanisms that provide detection of NM-MRI

Most of the efforts devoted to improving NM-MRI are to increase the contrast of the proton signal, in this case for NM, vs. the background signals. A phenomenon known as magnetization transfer (MT) is thought to provide the primary source of contrast in NM-MRI, due to the increased contrast found with MT preparation of NM-MRI pulse sequences.

MT contrast results from the interaction and exchange between water protons and the protons associated with macromolecules.^79,80^ Paramagnetic substances shorten relaxation times of both free protons and a pool of restricted macromolecule protons, and consequently the MT effect in the samples with paramagnetic ions is reduced.^81^

Quantitative MT is obtained by acquiring images at multiple radiofrequency offsets to generate an MT spectrum.^82^ From these, a model is fit^83^ to estimate indexes including the macromolecular to free pool size ratio, MT exchange rates, and the longitudinal and transverse relaxation times for each pool. At low offset frequencies, the radio frequency pulse will partially saturate the free pool through direct saturation (DS), and attenuate the observed signal.^84^ Thus, the observed signal attenuation is a combination of both MT and DS effects.

Iron affects MT spectra.^84^ While the main iron protein in human SN is ferritin, which possesses low paramagnetism, the main iron storage in pigmented SN neurons are NM–iron complexes, which are highly paramagnetic. This is because in the NM–iron paramagnetic complex (Fig. 6a), the chelating groups are catechols bearing a stable free radical, while in ferritin the chelating groups are amino acids. There are also aliphatic chains in NM that can further affect the exchange between macromolecular to free pool, and these aliphatic chains are absent in ferritin.^32^

With NM-MRI, the SNc, which contains the highest NM content, appears as an area of higher signal intensity than the SN pars reticulata (SNr), which is much lower in dopamine neurons and richer in iron^79,85,86^ but contains less NM–iron complex. Other iron-rich structures with lower NM content than SNc, such as putamen, globus pallidus, and caudate nucleus^56^, do not display the intense NM-MRI signal.

Multiple studies confirm that concentration-dependent T1 and T2 shortening are both much higher for the synthetic melanin–iron complexes than iron or metal-free melanin at the same concentrations.^87–89^ The melanin-iron structure contributes to an exceptional effect on T1 shortening that enhances its intensity in NM-MRI.^88^ A dominance of T1 over T2 effects in MT further contributes to SNc/gray matter contrast in NM-MRI. In summary, the contrast seen in NM-MRI is due to a combination of MT and T1 effects.^88^

## Initial in vivo MRI studies of NM

The first publication of in vivo human use of MRI to detect NM in SN and the LC was by Sasaki and colleagues, who used a *T*_1_ weighted protocol, but with a relatively short duration turbo “fast spin-echo” pulse sequence (TSE) with a 3 Tesla magnet: they wrote that 1.5 Tesla provided too little contrast.^90^ They named this approach “NM-sensitive MRI”. Consistent with Zecca and Swartz’s previous EPR data, the presence of the metals associated with NM pigment shortened the *T*_1_ signal.^39^

**Fig. 8:**
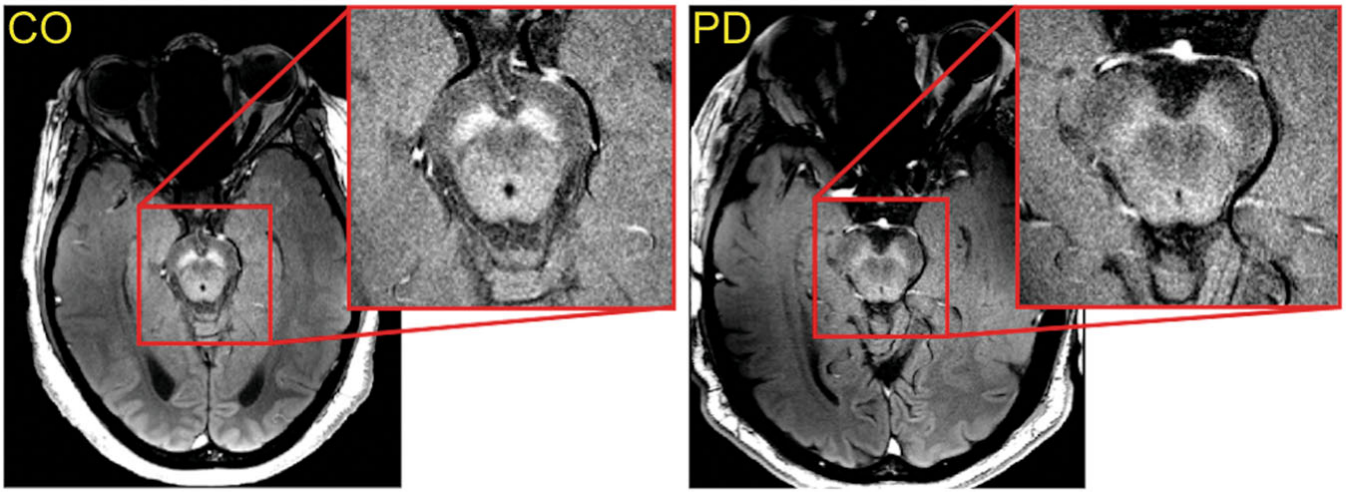
A comparison of SNc CNR in a control subject (labeled CO; left) and a PD patient (labeled PD; right). Both images were acquired using a NM-MRI sequence with an explicit MT preparation pulse^114^ and CNR was calculated using a standardized reference region placed in the cerebral peduncle.^121^ In both images, the red box shows a zoomed in view of the SNc. There is a 25% reduction in SNc CNR of the PD patient compared to SNc CNR of the control subject, indicating a loss of NM pigment in SNc of the PD patient. Identical windowing parameters were used to display both images

Following this initial report, TSE-based NM-MRI has since been used successfully (Fig. 8) to examine changes in SNc and LC arising from neuronal loss in PD.^90–94^

Kawaguchi and colleagues compared [^18^F]FE-PE2I PET, a radioligand for the DAT, with NM-MRI, and suggested that NM accumulation in the SN may depend on the intracellular dopamine concentration maintained by somatodendric DATs of each nigral dopamine neuron.^95^ Neuropathological findings in a subject with PD showed that NM-MRI signal intensity in the SNc is closely associated with the quantity of NM-containing neurons.^96^

These studies have only shown differences in NM-MRI signal between PD patients and control groups, and were not designed to evaluate disease status in individuals. In particular, the sensitivity and specificity of NM-MRI measurements to date does not provide differential diagnosis between PD, atypical parkinsonism,^93,97–100^ and essential tremor.^101^ This may be due not only to the NM-MRI technique but also to variability in multiple PD-like disorders.^102,103^ The remainder of this article is devoted to discussion of means to develop NM-MRI as an effective tool for diagnosis and measuring disease progression.

## Avenues to improve NM-MRI analysis

### Identification of regions of interest

Analysis of NM-MRI relies on a clear understanding of the anatomy of the SN^104,105^ and other dopaminergic (i.e., ventral tegmental area)^94^ or noradrenergic neurons (i.e., LC).^106,107^ The boundaries between the SNr and the SNc in humans are challenging to define, and clusters of SNc neurons are deeply embedded within the SNr. The ventral tegmental area moreover consists of a few heterogeneous groups of cells with also indistinct boundaries. The small size of the LC and its position make it difficult to image with 3 Tesla MRI and LC imaging may be better suited for ultra-high field (7 Tesla) MRI.^108^

Most studies of NM-MRI have used an approach in which SNc and a reference region are manually traced on a single axial slice from each subject’s NM scan. In some studies, the signal is averaged within each of these masks and then the contrast to noise ratio (CNR) is calculated according to the formula: ${\mathrm{CNR}} = \frac{{{{\rm{Mean}}\,{\rm{signal}}\,{\rm{SNc}}} - {{\rm{Mean}}\,{\rm{signal}}\,{\rm{Ref}}\,{\rm{region}}}}}{{{{\rm{Mean}}\,{\rm{signal}}\,{\rm{Ref}}\,{\rm{region}}}}}$, thereby providing a single CNR value for the SNc of each subject.^90,98^ Often, the CNR is reported separately for medial and lateral regions of SNc.^109–111^

While these methods show differences at the group level between PD patients and healthy controls, the approach has several limitations. It does not examine the entire SNc, but only a single slice. There is variability across and within studies of the location and orientation of this slice as well as in the placement of the borders of the SNc and reference regions within the slice. The borders of the SNc are difficult to define in individuals with PD, due to advanced loss of NM-containing neurons that decreases the contrast on the scan. This may bias the analysis by excluding highly impacted portions of the SNc, leading to an overestimate of the in-mask CNR of PD patients. There is thus a fundamental problem when the NM-MRI signal is measured on the same scan used to define the SNc regions of interest.

Recently developed SNc measurements avoid this circularity and have yielded good group separation, and, in some cases, provide a good correlation to illness severity.^94,109–111^ A drawback of SNc area measurements, however, is that an intensity threshold needs to be defined for a voxel to be considered within or outside the SNc. Valuable information regarding the intensity of the signal contrast is also lost in this dichotomization.

A related concern with the standard analysis approach is that it ignores subregional variation of NM-MRI signal within the SNc, although PD-related degeneration does not occur uniformly throughout this nucleus.^112^ Recent studies have taken this variation into account by dividing the SNc into two subregions based on overlap with other types of MRI contrast,^105^ or anatomical landmarks.^94,104^ There is currently little clear understanding of how to best perform such divisions with the SNc scans.

**Fig. 9:**
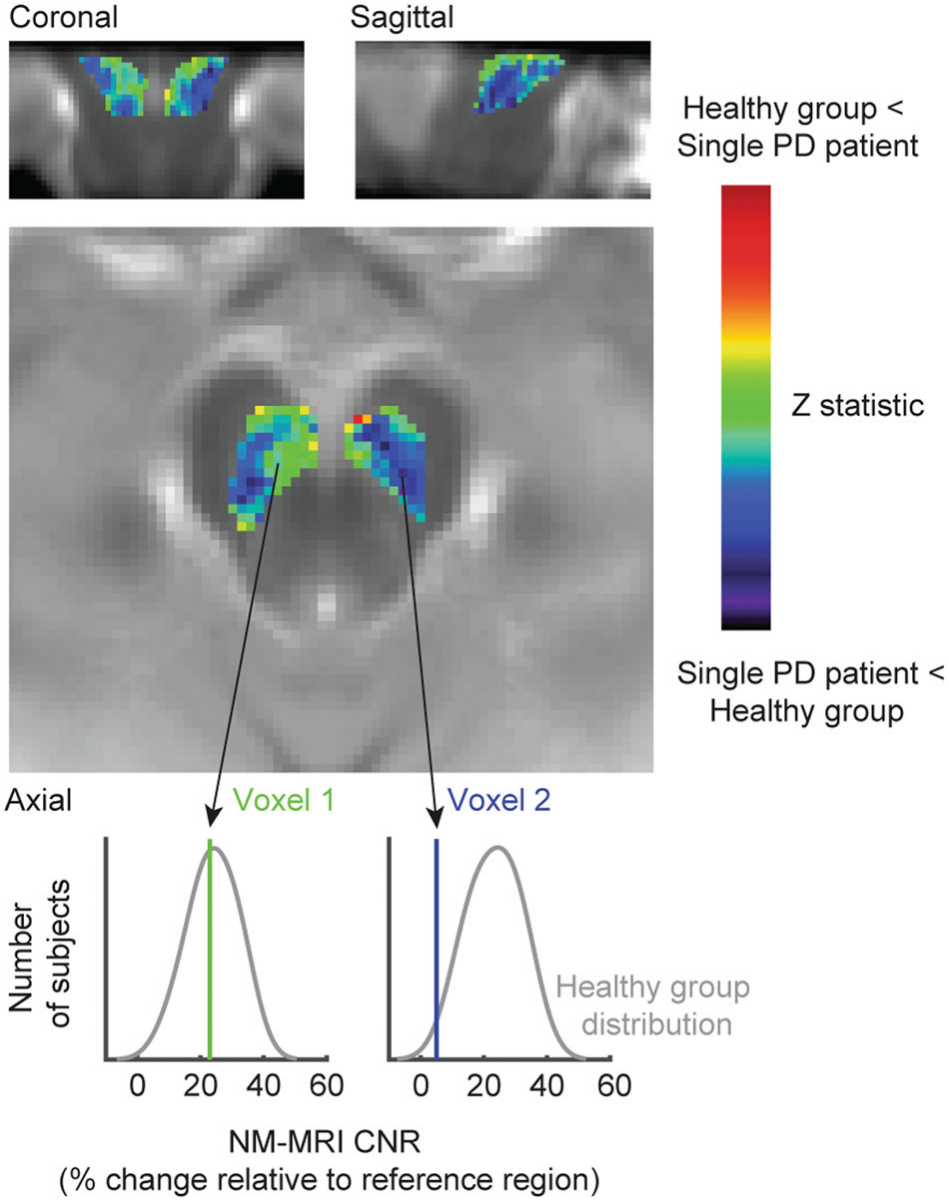
Voxelwise analysis showing data from a single PD patient relative to a group of matched healthy controls. Relative to the average of healthy individuals, many SNc voxels in this patient showed less NM-MRI signal (CNR; shown in blue) but some voxels showed similar or higher CNR (green to red). Note that a medial/lateral division of SNc would not fully separate the most impacted regions for this patient. At bottom, smoothed histograms indicate distribution of CNR in the group of healthy controls for two different SNc voxels. The CNR of each voxel in this patient is indicated by a vertical line overlaying each histogram; arrows show the location of each voxel on the axial plane. According to the voxel plotted at the right, this patient has a very low probability of belonging to the control group; however, as seen from the voxel at left, this patient’s CNR is well within the healthy range

Analysis of the NM-MRI signal in a voxelwise manner more fully captures fine-grained anatomical information contained in the images. Such an approach can identify precisely which subregions of SNc are most impacted in PD and promise to improve classification accuracy by focusing on subregions rather than averaging the signal across impacted and spared subregions. Horga and collaborators have implemented a voxelwise analysis^113^ by normalizing NM-MRI scans into Montreal Neurological Institute standard brain space based on tissue segmentation from T1-weighted and T2-weighted structural scans. This approach does not use subject-specific masks of the SNc or the reference region but rather an atlas mask created from the average of a large dataset of NM-MRI scans from healthy controls. The voxelwise approach has proven effective for various applications of NM-MRI (see Fig. 9). Future work might further capitalize on regional variation via multivoxel pattern analysis (e.g., support vector machine or regularized regression techniques).

### Improvement of NM-MRI signal acquisition

Improvements to NM-MRI can be made in image acquisition and processing techniques to better delineate SNc or LC and raise reproducibility. For example, the TSE sequence relies on incidental MT effects from a multi-slice interleaved acquisition to image the NM structures.^79,80^ This approach is not as efficient in generating NM-sensitive contrast as explicit MT preparation pulses.^114^ In addition, the TSE approach uses a large amount of radio frequency energy, and MRI scanner safety limits may ask the operator to reduce the number of slices acquired or require other changes in scan parameters, such as lengthening repetition time or reducing the echo train length.^114^ A decreased number of acquired slices may compromise the ability of the sequence to acquire LC and SNc in a single acquisition. Changes in acquisition parameters may further limit the reproducibility of NM-sensitive contrast by adding confounding factors to group analyses.

Individual NM-MRI images have a low signal to noise ratio (SNR), necessitating a scan with multiple measurements and a long acquisition time. The choice of MRI pulse sequence may also affect sensitivity to motion artifacts. As 3D MRI pulse sequences have longer acquisition times than 2D sequences, 3D acquisitions are more prone to motion artifacts. This can be particularly challenging in studies of populations with motor disorders, particular for the LC, which is a much smaller region than the SN.

### Improvement of NM-MRI signal analysis

As detailed in the section above, “Changes in NM and iron molecular content during normal aging and PD”, NM accumulates with age. While there should be a means to factor age in statistical analyses, there is no consensus on how to do so. Existing data from large cross-sectional studies show that the average NM-MRI signal in the entire SN increases with age in healthy individuals,^115,116^ although this was not observed in some small studies.^90,97,117,118^ Factors underlying this discrepancy could include subregional variability in age-related changes in the SNc NM-MRI signal (requiring detection by anatomically precise methods) or non-linearity in age-related changes if the signal declines in some fraction of individuals with advanced age. Indeed, both of these observations have been reported in investigations of age-related changes of the LC using NM-MRI.^119,120^ At this time, it remains unclear if age is generally better fit to linear or non-linear models, whether age-related changes occur uniformly throughout the SN, or whether age-by-diagnostic–group interactions should be considered.

Some of these questions could be clearly addressed if data were available from a large NM-MRI study of healthy individuals sampled longitudinally across the full range of lifespan. Image processing techniques can improve delineation of NM containing structures by reducing motion artifacts. The images from each measurement can be saved and corrected for motion offline and then averaged. Processing can also increase the CNR. In particular, the use of reference regions of interest selected in a standard space and transformed to native space removes variation.

LC volume and NM contrast exhibit high test-retest reproducibility in both single day rescans^121^ and 2.8 month follow-up rescans.^122^ LC biomarkers are slightly more reproducible in a NM-MRI sequence using explicit MT-effects than in NM-MRI from the TSE sequence.^121,122^ The increased reproducibility of the MT-based NM-MRI approach may be due to a combination of increased NM contrast from explicit MT effects,^114^ automated processing using standard space regions of interest used in the explicit MT NM-MRI analysis^121^ vs. a manual analysis,^122^ and the time interval between scans. In SNc, volume and MT contrast, as measured from explicit NM-MRI, exhibited nearly perfect reproducibility in interscan measurements.^121^

## Discussion of NM-MRI as a biomarker for PD

An important goal for the development of NM-MRI is to establish its correlation with the clinical status of PD. Conclusions from current studies are controversial, with some reporting a correlation with the UPDRS motor scale,^94,95,104,105^ and others not.^123^ One study reported a reduction of SNc area of ~17.5% per year in people with PD (over 2.3 ± 1.1 years of follow-up),^92^ which is slightly higher than studies on nigrostriatal dopaminergic degeneration (i.e., 6–13%).^124,125^ It has been claimed that striatal dopamine loss is related almost exclusively to bradykinesia.^124^ Carriers of *parkin* or *LRRK2* mutations and individuals with RBD have been investigated, and those with PD showed a comparable SNc volume as idiopathic PD,^111,123^ which is in line with a similar reduction of nigrostriatal dopaminergic innervation in these two disease entities.^126^ A loss of dorsolateral nigral hyperintensity was described in about two-third of individuals with RBD,^127^ together with reduced signal intensity in the coeruleus/sub-coeruleus complex.^106^ We suggest that a major concern with these studies is that they mostly neglect the increase of NM with age as detailed in the section above, “Changes in NM and iron molecular content during normal aging and PD”.

Another important goal is to cross-validate NM-MRI with other imaging approaches. One approach would be to correlate NM-MRI with DAT levels. Bae and colleagues reported a concordance rate of 86.2% between susceptibility-weighted imaging and the density of DAT in the striatum, measured with 123I-2β-carbomethoxy-3β-(4-iodophenyl)-N-(3-fluoropropyl)-nortropane SPECT.^100^ Further studies confirmed a good correlation between striatal DAT and NM-sensitive, but not iron-sensitive MRI, images.^107,109^ The role of age is relevant for these studies, as it influences both NM concentration and striatal DAT density.^128^ Signal intensity ratios in the medial and lateral SNc on NM-MRI positively correlated with heart-to-mediastinum count ratios on ^123^I-metaiodobenzylguanidine (MIBG) SPECT,^129^ another substrate for catecholamine transporters, although it should be noted that MIBG SPECT is not a sensitive marker of early PD and does not correlate with PD stages.^130^ Indeed, the combination of the NM-MRI and MIBG SPECT showed poor sensitivity and specificity when discriminating subjects with Alzheimer’s disease and PD patients at an early stage (H&Y I-II).^129^

Another important candidate for cross-validation of NM-MRI with another imaging approach is [^18^F]-AV-1451, which was developed for high affinity to neurofibrillary tau in Alzheimer’s disease but also binds to NM and melanin.^131,132^ This probe has now been used to examine PD patients,^133^ including in studies attempting to differentiate the tauopathy progressive supranuclear palsy from PD,^134,135^ and cross-validation studies with NM-MRI and this tracer may help characterize the features of both approaches.

A relevant issue is whether L-DOPA administration, which as discussed can produce NM in neuronal cultures,^22^ also enhances NM in PD patients. This question has to date not been examined.

Another factor that may impact the NM-MRI signal is variation in genes affecting NM accumulation. Expression of VMAT2 strongly impacts NM accumulation^22,29^ (see the section above, “NM pigment is synthesized from oxidized catecholamines that are trapped within autophagic organelles”). It would be valuable to test whether NM-MRI signal correlates to VMAT2 binding (based on PET imaging data) and whether controlling for this could improve the ability of NM-MRI to track clinical measures. Although PET imaging in support of NM-MRI would be impractical for most applications, perhaps VMAT2 gene variants could be identified that impact NM-MRI signal, providing a practical covariate in NM-MRI analysis.

## Recommendations for clinical use

An effective application of NM-MRI as a biomarker for PD will be based on multiple factors. The fundamental issue is the ability to distinguish changes in an individual with age. The measurements by Zecca et al.^15,41^ on autopsy tissue indicate that the concentration of NM pigment in normal SN increases by about 50% between the ages of 40 and 60: an increase or loss of signal during this period in comparison with the typical change should indicate if the SN is aging normally or if degeneration has begun.

The high test-retest reproducibility of NM-MRI techniques makes longitudinal studies of NM possible, and such studies are critical to the development of this approach. To date, however, studies examining PD-related NM-MRI have examined differences in cross-sectional populations. Longitudinal studies will provide information on the timing of PD-related changes in LC and SN, as well as developing novel biomarkers for clinical diagnosis of PD, and for use as an outcome measure in clinical trials. Current measures evaluating efficacy of therapeutic treatment are based on how well the treatment mitigates symptoms, and not on how the treatment arrests degeneration or regeneration in affected structures including LC or SNc.

The voxelwise method of NM-MRI analysis is now fully automated and can be run without requiring expertise or staff time, minimizing impediments to its wide-scale use as a routine clinical and screening tool. As long as the scans are manually checked for motion artifacts and stack placement, the scan image can be taken directly from the scanner and processed by software to generate statistics and a brain map (similar to Fig. 9), reflecting SNc signal loss in an individual PD patient. With voxelwise analysis and current techniques that require only a few minutes to conduct, longitudinal changes should be measurable over intervals of a few years.

MRI is often used as a routine clinical test for PD to rule out other contributing etiologies. Adding a MRI scan sequence for NM detection could be done without extending scan time significantly. This may be particularly important for those who have genetic or environmental factors that may increase the risk for PD.

An interesting direction will also be to measure the loss of NM in LC, as this is widely suspected to occur prior to SN loss.^136^ There is also a linear increase in NM content in the LC with age (Fig. 7b), although total iron concentration remains stable and lower than that of SN (Fig. 6b).^41^ Interestingly, two NM-MRI studies on PD patients report that low levels of LC NM are present specifically in those with RBD,^117,137^ and this is consistent with suggestions that abnormal muscle tone during rapid eye movement is associated with loss of LC NE or nearby neurons. A concern for PD diagnosis, however, is that the LC neurons are also lost in Alzheimer’s and other disorders,^14^ and so an LC assay may be less specific for PD. A new approach for MRI detection of both SN and LC during a single MRI session^114^ may offer a useful approach. Whether LC signal differences between multisystem atrophy and PD may differentiate them^97,98^ or the pattern of reduction of NM in SN is different in atypical parkinsonisms (e.g., progressive supranuclear palsy and multisystem atrophy) that also feature degeneration of the SN dopaminergic neurons,^93,97^ are controversial. Whether a voxelwise approach to examine regions of loss within the SNc or the time course of the loss of NM in the various parkinsonian conditions may reveal differential patterns or rate of loss in various parkinsonisms needs to be studied more systematically in a larger cohort.

Additional biomarkers such as immune-related signals or PET and SPECT imaging of dopamine-related markers, in conjunction with NM-MRI, may further contribute to the accuracy of diagnosis and measure disease stages and responses to therapies.

We propose that a worthwhile goal may be to analyze individuals every 10 years beginning with a baseline at 40 to compare the normal increase in signal with degeneration that might become detectable by age 50 or later. For those identified for possible presymptomatic PD, such as individuals with PD-associated *GBA*, *LRRK2*, and *parkin* alleles or those with RBD, more frequent measurements may detect if protective therapies, many of which are currently in development and some of which will be more effective for particular PD subtypes and patients, are effective for that patient.
